# Association between cardiopulmonary resuscitation audit results with in-situ simulation and in-hospital cardiac arrest outcomes and key performance indicators

**DOI:** 10.1186/s12872-023-03320-w

**Published:** 2023-06-13

**Authors:** Onlak Ruangsomboon, Usapan Surabenjawongse, Pongthorn Jantataeme, Thanawin Chawaruechai, Khemchat Wangtawesap, Tipa Chakorn

**Affiliations:** 1 Department of Emergency Medicine, Faculty of Medicine, Siriraj Hospital, Mahidol University, 2 Wanglang Road, Bangkoknoi, Bangkok, 10700 Thailand; 2 Siriraj Medical Simulation for Education and Training (SiMSET), Faculty of Medicine, Siriraj Hospital, Mahidol University, Bangkok, Thailand; 3 Department of Anesthesiology, Faculty of Medicine, Siriraj Hospital, Mahidol University, 2 Wanglang Road, Bangkoknoi, Bangkok, 10700 Thailand

**Keywords:** In-hospital cardiac arrest, Audit CPR, CPR outcome, In-situ simulation, Simulation

## Abstract

**Introduction:**

In-situ simulation (ISS) is a method to evaluate the performance of hospital units in performing cardiopulmonary resuscitation (CPR). It is conducted by placing a high-fidelity mannequin at hospital units with simulated scenarios and having each unit’s performance evaluated. However, little is known about its impact on actual patient outcomes. Therefore, we aimed to evaluate the association between the ISS results and actual outcomes of patients with in-hospital cardiac arrest (IHCA).

**Methods:**

This retrospective study was conducted by reviewing Siriraj Hospital’s CPR ISS results in association with the data of IHCA patients between January 2012 and January 2019. Actual outcomes were determined by patients’ outcomes (sustained return of spontaneous circulation (ROSC) and survival to hospital discharge) and arrest performance indicators (time-to-first-epinephrine and time-to-defibrillation). These outcomes were investigated for association with the ISS scores in multilevel regression models with hospital units as clusters.

**Results:**

There were 2146 cardiac arrests included with sustained ROSC rate of 65.3% and survival to hospital discharge rate of 12.9%. Higher ISS scores were significantly associated with improved sustained ROSC rate (adjusted odds ratio 1.32 (95%CI 1.04, 1.67); *p* = 0.01) and a decrease in time-to-defibrillation (-0.42 (95%CI -0.73, -0.11); *p* = 0.009). Although higher scores were also associated with better survival to hospital discharge and a decrease in time-to-first-epinephrine, most models for these outcomes failed to reach statistical significance.

**Conclusion:**

CPR ISS results were associated with some important patient outcomes and arrest performance indicators. Therefore, it may be an appropriate performance evaluation method that can guide the direction of improvement.

**Supplementary Information:**

The online version contains supplementary material available at 10.1186/s12872-023-03320-w.

## Introduction

In-hospital cardiac arrest (IHCA) is a deleterious condition that results in a high mortality rate and poor neurological outcomes. [1, 2] Therefore, continuous quality assessment and improvement are mandatory to improve patients’ outcomes. However, cardiopulmonary resuscitation (CPR) is a procedure that requires a multidisciplinary team collaboration; thus, the measures for performance evaluation need to be comprehensive, and their processes are often complex. Hospital inspections and their published ratings have been employed as a tool to measure and improve hospital performance. [3] Nonetheless, their impacts on the quality of care have been varied, and results have conflicted between studies. [3–5].

Simulation-based training, which has now become a popular technique in medical education, is the core of CPR education and training based on the American Heart Association (AHA) and European Resuscitation Council (ERC) curriculums. [6–10] The CPR center at Siriraj Hospital, Thailand, has also integrated the technique into advanced cardiovascular life support (ACLS) and basic life support (BLS) training for our healthcare personnel. Moreover, the Siriraj CPR center has implemented a tool to evaluate CPR performance based on the inspections and rating principle called “the CPR audit”. The audit employed in-situ simulation, whereby a high-fidelity manikin is placed in the hospital unit where an actual IHCA event can occur with a cardiac arrest scenario simulated. The performance of the resuscitation by each unit is then observed and rated by auditors. This technique has been shown to provide more benefits than the traditional simulation at the training center because it could more accurately reflect real situations with available resources in each area. [11–13] However, little is known about its value as an evaluation tool of CPR performance on actual IHCA outcomes of cardiac arrest patients.

Therefore, this study aimed to evaluate whether CPR in-situ simulation performance was associated with actual IHCA CPR outcomes.

## Methods

### Study setting

This retrospective observational study was conducted in Siriraj Hospital, an academic, tertiary care hospital in Bangkok, Thailand, with approximately 2,400 inpatient beds, 12 intensive care units (ICU), and 2 emergency departments (ED) including trauma and non-trauma EDs. The study was approved by the Siriraj Institutional Review Board (certificate number 727/2020), and informed consent was waived. The study was performed in accordance with the Good Clinical Practice (GCP) guideline and the Declaration of Helsinki, and reported according to the STROBE standards. [14].

### Objectives

The primary aim of the study was to assess whether CPR in-situ simulation results were associated with actual IHCA CPR outcomes, sustained ROSC and survival-to-hospital-discharge. The secondary aim was to assess whether CPR in-situ simulation results were associated with important arrest performance indicators, time-to-first-epinephrine for initial non-shockable rhythm and time-to-defibrillation (see the outcome definitions in Additional File 1).

### In-situ simulation CPR audit

**Fig. 1:**
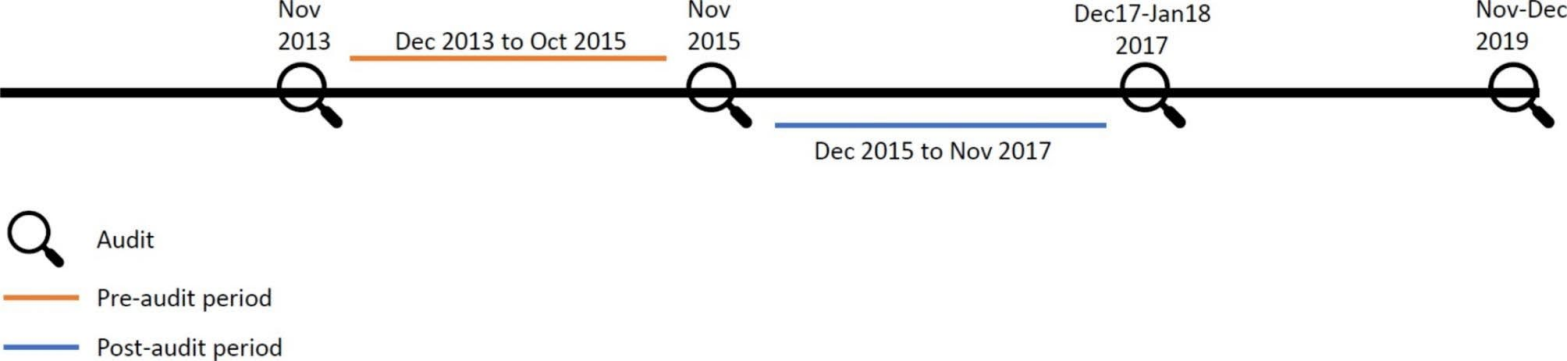
Conceptual diagram of time for in-situ simulation audit over the study period

The Siriraj CPR center is responsible for providing CPR training for the hospital’s personnel and conducting the CPR audit for all hospital units/wards. These audits were conducted on alternative years starting from 2009. Four previous audit cycles took place over one or two months in each of the years 2013, 2015, 2017 and 2019 (Fig. 1). For each audit cycle, each hospital unit underwent one in-situ simulation scenario.

The CPR center committee categorized the hospital units into five unit-categories according to their risk of having an occurrence of IHCA; (1) ED and ICU, (2) critical wards, (3) procedural units, (4) general wards, and (5) outpatient units (OPD). In unit-category 1, EDs and ICUs, physicians and nurses are available on the floor 24 h. In critical wards, the ratio of physicians and nurses per patient was lower than in the EDs and ICUs. The first two unit-categories have their own emergency response systems, while the other three unit-categories were covered by an on-call hospital ACLS team who would respond to the emergency activation system of the hospital. All physicians and nurses in unit-category 1 through 3 are obligated to attend an ACLS course organized by the Siriraj CPR center. They also have to renew the course every three years. While the nurses in unit category 4 and 5 have to at least pass a BLS course. The full details of each unit-category and their emergency response systems can be found in the supplementary material (Additional file 2).

#### The audit process

During office hours on random dates within the pre-announced period, a high-fidelity mannequin was transported to the audited units. An unannounced simulation scenario was randomly chosen by the auditors on the audit date for each hospital unit. The scene was videotaped, and the time and quality of each intervention were recorded by using the program embedded in the mannequin. After the scenario, the auditors provided verbal feedback to each audited team during a debrief. Also, the audited teams later received written reports on their evaluations and suggestions for improvement. If they failed an audit, further simulation team and individual training was provided by the CPR center.

#### The audit scenarios

A pool of 6–8 scenarios, all of which had at least one shockable rhythm included, for each hospital unit type was created by the lead auditors prior to each audit cycle. These scenarios were simulated by a group of blind auditors and modified as appropriate to assure their appropriateness and equivalence in difficulty for the hospital unit type. Each scenario was allowed to run for 8 min. The algorithms for the scenarios were pre-programmed into the high-fidelity mannequins to ensure that they ran the same way should the same scenario be selected for different hospital units.

#### The audit evaluation tool and evaluation process

The CPR center committee, who were the lead auditors during the first years of the audit development, developed the CPR audit checklist for evaluation in accordance with the AHA and ERC guidelines. The checklist was revised in 2011 and 2015 to comply with the 2010 and the 2015 guidelines. [10, 15, 16] It included all the recommendations for effective resuscitation based on the referenced guidelines. Details of the tool can be found in Additional file 3. The audited team was evaluated in 4 domains (score range 0–10 for each domain); (1) first response reaction, (2) overall ACLS performance, (3) quality of team dynamics, and (4) Emergency Activation System (EAS) performance. Before the audit in 2013, the tool was tested for its inter-rater reliability among a sample of 20 auditors. Rated scores of all the domains had inter-rater reliability of > 0.8.

Each auditor independently rated the audited team’s performance in real time. On the same day of the audit, they re-watched the videotaped scene together and decided on the final score for each domain based on the average score of all auditors or by consensus. The passing criterion was an overall score in 4 domains of at least 60% weighted as 30% first response reaction, 30% ACLS performance, 30% team dynamics, and 10% EAS performance. The weightings were determined by the CPR center committee based on their importance in delivering effective CPR and successful outcomes.

#### The auditors

All auditors were licensed ACLS instructors consisting of registered nurses and physicians (emergency physicians, anesthesiologists, and internal medicine physicians). Before becoming auditors, they underwent a one-day training session. The session included the audit details and process, the scenarios, the evaluation tool, and the general guide for evaluation and debriefing. At the end of the session, potential auditors underwent simulation and written evaluation before being approved as validated auditors. The total number of validated auditors increased from 25 in 2013 (48% were physicians) to 41 in 2019 (51.2% were physicians). At least three validated auditors, one of which had to be a physician, were present at each in-situ simulation.

#### The audited teams

To best simulate real-life situations, the auditors did not pre-specify or limit the number of team members allowed in the audit scenarios. Healthcare providers of any profession (i.e., physicians, registered, and practical nurses) and at any level of experience who were responsible for the hospital unit could participate. Performance was rated as a team, and the evaluation was not adjusted for the number of team members, their professions, or their experience in CPR and in-situ simulations.

Participants in the audit were ensured of their confidentiality should the audit results be analyzed for research purposes, as no identifiable data to a specific hospital unit or individual participant would be reported. Also, video recordings of the audit scenarios were deleted after each cycle’s analysis unless permission was given by the participants to keep them for training purposes.

### Actual IHCA patient population, data collection, and outcomes

Patients aged at least 15 years old with an IHCA and resuscitation in audited units were included. This age cut-point was determined based on the minimum age of patients treated in the participating hospital units, all of which were adult units. Cardiac arrest was defined as a delivery of chest compressions and/or defibrillation. [17] We included data of all cardiac arrests that occurred in the 5 unit-categories and referred to them as IHCA to correspond to the audit system. Data of IHCA patients admitted between January 2012 and December 2018 with cardiac arrest events occurring between January 2012 and January 2019 were reviewed.

CPR data were collected from the CPR record form designed by the CPR center and was universally used throughout the hospital. All units had to send this form back to the CPR center within 24 h after an IHCA. Data recorded were patient details, CPR process, and immediate outcome after resuscitation. The CPR center later collected the outcome at discharge from medical records. Definitions of the variables were according to the Utstein Resuscitation Registry Template for IHCA. [17] The primary patient outcome was sustained return of spontaneous circulation (ROSC), defined as palpable pulse or systolic blood pressure ≥ 60 mmHg for ≥ 20 min. The secondary patient outcome was survival-to-hospital-discharge.

### Measuring the association between audit results and actual outcomes

To answer the study’s main objective, we analyzed the data by associating the audit scores with actual IHCA outcomes during the pre-audit period, defined as the duration from the previous audit date to the index cycle audit date (Fig. 1), as we hypothesized that hospital units that can deliver favorable patient outcomes and arrest performance indicators should also acquire high audit scores when evaluated (hypothesis 1). The pre-audit period was employed for the analysis as opposed to the post-audit period because we considered the CPR in-situ simulation as an evaluation tool rather than a training intervention.

As a further analysis, we assessed whether hospital units failing an in-situ simulation (overall score < 60% passing criteria) improved their outcomes and arrest performance indicators after the audit by comparing their actual outcomes occurring during the pre-audit period (same definition as above) to those of the post-audit period (the duration from the index audit date to the date of the next audit cycle) (Fig. 1). We hypothesized failing units should improve their performance afterwards (hypothesis 2). Moreover, we evaluated whether the units that passed an audit could further improve the study outcomes.

The audit cycles of the years 2013, 2015, and 2017 had available pre- and post-audit data. However, we only analyzed associations based on the in-situ simulation results surveyed in 2015 and their related pre-audit and post-audit durations (Fig. 1). This was because, in the 2013 audit cycle, 14.8% of hospital units did not participate, and in the 2017 audit cycle, there was gross imbalance in pre- and post-audit periods of observation. The 2015 audit cycle included all hospital units and had relatively equal pre- and post-time durations, thereby having relatively balanced time periods to accumulate IHCA events to be compared. Accordingly, only actual IHCA patients who had cardiac arrest during the pre- and post-audit durations of the audit cycle 2015 was included in the primary analysis (Fig. 1).

### Statistical analyses

Descriptive statistics are presented for patient characteristics, outcomes, and the audit results. We used one-way ANOVA to compare audit scores by hospital unit-category in each audit cycle. We collapsed the critical and general wards categories into “ward” as well as procedural and OPD into “other” because of very few ROSC events in general wards and OPDs.

For the primary hypothesis, we compared outcomes by audit score using a series of cross-sectional multilevel regression models. For each study outcome, we first obtained intraclass correlation coefficient to evaluate the extent to which outcomes could be explained by clusters or hospital units. [18] Consequently, two models were constructed. [19] For sustained ROSC and survival-to-hospital-discharge, we used multilevel logistic regression models. Model 1 fitted audit score with the arrest unit to estimate the crude odds ratio (cOR). Model 2 also included the arrest unit type (ED, ICU, ward or other) and patient-level variables that may contribute to the process of sorting individuals into hospital units, [20] which are age, sex, initial shockable rhythm, end-stage renal disease, chronic kidney disease, hematologic malignancy, solid neoplasia, heart disease, and liver disease, to estimate an adjusted odds ratio (aOR) for audit score adjusted for both hospital unit- and patient-level confounders. For time-to-first-epinephrine and time-to-defibrillation, we employed multilevel linear regression models. All models were constructed in the same manner as the logistic models except that only one patient-level confounder was used in time-to-first-epinephrine and time-to-defibrillation models, namely pre-arrest intravenous access and pre-arrest electrocardiogram monitoring, respectively. For the secondary hypothesis, we also employed multilevel logistic models for ROSC and mortality outcomes and linear models for two other outcomes with each audited unit as a cluster to compare changes in the study outcomes between pre- and post-audit periods at the hospital unit level. Only hospital units with arrests in both pre- and post-audit periods were included.

Moreover, we conducted two sensitivity analyses. The first excluded data in the “ED” and “other” unit type as per the Get-With-the-Guidelines’s definition of IHCA, a more commonly-known definition compared to ours, which was adapted to correspond to our audit system. [15] The second excluded cardiac arrests occurring within the one month prior to the audit to exclude any “anticipation effect” during the pre-audit period for the primary hypothesis and the six months after the audit to allow “time for implementation of improvements” during the post-audit period for the secondary hypothesis. We also performed subgroup analyses for the two hypotheses by unit type, but “other” was not analyzed because of sparse data. For the secondary hypothesis, ED was not analyzed because ED never failed an audit.

We performed a complete case analysis. Only the data of the audit cycle 2015 were analyzed in the multilevel regression models. Unbiased estimation of level-2 effects in multilevel models requires at least 50 clusters and 2 observations per cluster. [21, 22] Thus, we consider any models with ≥ 50 clusters (hospital units) to be confirmatory. We used R software version 4.5.0 (R Foundation for Statistical Computing, Vienna, Austria) using the lme4 package. A p-value of < 0.05 was considered significant.

## Results

### CPR in-situ simulation results

In 2013, 85.2% of hospital units participated in the audit whereas all units participated in the following audit cycles. The proportion of units passing the 60% criterion decreased from 86.1% to 2013 to 49.5% in 2019, corresponding to lower mean scores of later audit cycles (Table 1). Among unit types, ED and ICU had the highest mean scores in all four audit inspections while OPD had the lowest mean scores in the last three audits.

**Table 1:** Overall audit scores by year of audit (score range 0–10)

Arrest unit type	Audit year 2013	Audit year 2015	Audit year 2017	Audit year 2019
All	7.27 ± 1.52 (n = 144)	6.56 ± 1.54 (n = 177)	6.26 ± 1.72 (n = 199)	5.78 ± 1.78 (n = 212)
ED and ICU	8.14 ± 0.97 (n = 14)	7.78 ± 0.93 (n = 14)	7.0 ± 1.10 (n = 14)	6.71 ± 1.65 (n = 14)
Critical ward	7.71 ± 0.88 (n = 34)	6.74 ± 1.01 (n = 33)	6.37 ± 1.63 (n = 38)	5.65 ± 1.54 (n = 51)
General ward	7.48 ± 0.76 (n = 32)	6.60 ± 1.30 (n = 44)	6.40 ± 1.36 (n = 42)	5.41 ± 1.74 (n = 37)
Procedural unit	6.67 ± 1.82 (n = 31)	6.32 ± 1.75 (n = 32)	6.15 ± 2.0 (n = 45)	6.25 ± 1.75 (n = 49)
OPD	6.81 ± 2.10 (n = 33)	6.23 ± 1.82 (n = 54)	6.0 ± 1.91 (n = 60)	5.52 ± 1.93 (n = 61)
P-value	0.003	0.013	0.288	0.033
ED	8.85 ± 1.10 (n = 2)	8.0 ± 0.42 (n = 2)	8.15 ± 0.49 (n = 2)	8.0 ± 1.41 (n = 2)
Units with score > 60%	124 (86.11%)	125 (70.62%)	127 (63.82%)	105 (49.53)

Notes:- Data are presented as mean ± SD or frequency (%). Arrest unit types were compared by one-way analysis of variance. A p-value of < 0.05 was considered significant

Abbreviations: ED, emergency department; ICU, intensive care unit; OPD, outpatient unit

### Actual CPR outcomes

Between January 2012 and January 2019, 2146 patients had IHCA in Siriraj Hospital. Their median age was 68 years; 45% were female. Of these, 65.3% had sustained ROSC, and 12.9% survived to discharge (Table 2). Most initial rhythms (84.4%) were non-shockable. Approximately half of the events occurred in the ED or ICU. These hospital unit types were generally associated with better patient outcomes than the other types (Additional file 4). Missing data are reported in the footnote of Table 2.

**Table 2:** Patient characteristics

Characteristic	All patients (N = 2146)	Pre-audit 2015 (N = 483)	Post-audit 2015 (N = 324)
**Demographics**			
Age, median (IQR), y	68 (54, 78)	65 (53, 77)	67 (57, 79)
Female	965 (45.0)	210 (43.5)	150 (46.3)
**Pre-existing conditions**			
Diabetes mellitus	678 (31.6)	156 (32.2)	94 (29.0)
Hypertension	1118 (52.1)	263 (54.5)	136 (50.3)
Chronic kidney disease	473 (22.0)	116 (24.0)	67 (20.7)
End-stage renal disease	45 (2.1)	11 (2.3)	6 (1.9)
Cancer			
- Non-metastatic	105 (4.9)	26 (5.4)	17 (5.2)
- Metastatic/advanced	16 (0.8)	3 (0.6)	2 (0.6)
Hematologic malignancy	44 (2.1)	6 (1.2)	14 (4.3)
Old stroke	62 (2.9)	16 (3.3)	14 (4.3)
Heart disease	481 (22.4)	123 (25.5)	62 (19.1)
Liver disease	167 (7.8)	30 (6.2)	28 (8.6)
Chronic obstructive pulmonary disease	60 (2.8)	14 (2.9)	10 (3.1)
^a^Consciousness			
- Alert	562 (27.2)	137 (28.4)	80 (24.7)
- Confused	143 (6.9)	35 (7.2)	22 (6.8)
- Stupor	310 (15.0)	85 (17.6)	42 (13.0)
- Semi-coma	184 (8.9)	44 (9.1)	24 (7.4)
- Coma	864 (41.9)	171 (35.4)	153 (47.2)
**Interventions in place at time of arrest**			
Any intervention	1761 (82.1)	448 (92.8)	264 (81.5)
Mechanical ventilation	1249 (58.2)	276 (57.1)	180 (55.6)
Electrocardiogram monitoring	1306 (60.9)	294 (60.9)	192 (59.3)
Intra-arterial catheter	389 (18.1)	70 (14.5)	52(16.0)
Intra-venous catheter	1564 (72.9)	356 (73.7)	235 (72.5)
Pacemaker	67 (3.1)	19 (3.9)	6 (1.9)
Implantable defibrillator	10 (0.5)	3 (0.6)	1 (0.3)
Hemodynamic drug support	21 (1.0)	3 (0.6)	1 (0.3)
**Arrest characteristics**			
Type of hospital unit			
- Emergency department	384 (17.9)	70 (14.5)	(32.3)
- Intensive care unit	681 (31.7)	143 (29.6)	(26.2)
- Ward	1052 (49.0)	263 (54.5)	(39.8)
- ^b^Other	29 (1.4)	7 (1.4)	7 (2.2)
Time of arrest			
- Morning shift	763 (35.6)	174 (36.0)	116 (35.8)
- Afternoon shift	741 (34.5)	162 (33.5)	112 (34.6)
- Night shift	642 (29.9)	146 (30.2)	96 (29.6)
^c^Witnessed	2102 (98.1)	483 (100)	322 (99.4)
^d^Initial rhythm			
- VF/pulseless VT	311 (15.7)	73 (15.1)	45 (13.9)
- Pulseless electrical activity	1151 (58.0)	254 (52.6)	205 (63.3)
- Asystole	523 (26.4)	139 (28.8)	70 (21.6)
**Arrest outcomes**			
Return of spontaneous circulation ≥ 20 min	1402 (65.3)	316 (65.4)	201 (62.0)
Survival-to-hospital-discharge	276 (12.9)	54 (11.2)	56 (17.3)
Survival-to-hospital -discharge with CPC scale = 1	33 (1.5)	9 (1.9)	11 (3.4)
^e^Time-to-first-epinephrine for non-shockable initial rhythm, median (IQR), min	1 (0, 3)	1 (0, 3)	1 (0, 3)
^f^Time-to-defibrillation for initial shockable rhythm, median (IQR), min	3 (1, 5)	3 (1, 8)	2 (1, 5)

Notes:- Data are presented as n (%) unless stated otherwise

^a^missing data n = 83, ^b^Others includes procedural units and outpatient department, ^c^missing data n = 3, ^d^missing data n = 161

^e^n=1571 analyzed excluding events with time-to-first-epinephrine before time of no pulse, the presence of pre-arrest hemodynamic supportive agent, and unknown initial rhythm, ^f^n=188 analyzed excluding events with time of first defibrillation before time of no pulse, the presence of pre-arrest hemodynamic supportive agent, the presence of pre-arrest implantable defibrillator, and unknown initial rhythm

Abbreviations: CPC, Cerebral Performance Category; IQR, interquartile range; VF, ventricular fibrillation; VT, ventricular tachycardia

**Fig. 2:**
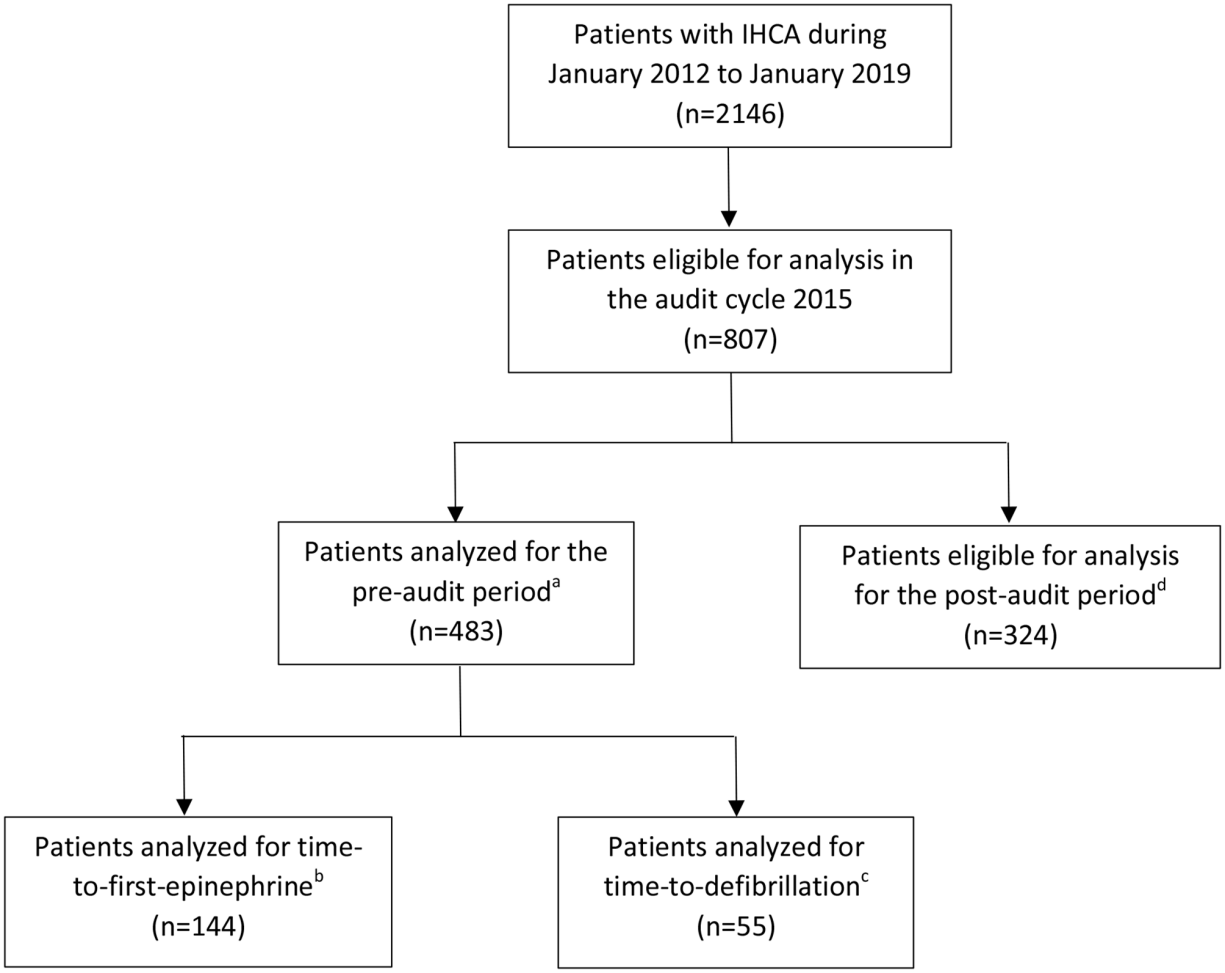
**Patients’ flow chart** Notes:- ^a^The pre-audit population was included in the both the analysis of the pre-inspection period and the pre-post analysis. For the pre-inspection analysis, the whole 483 patients were analyzed for return of spontaneous circulation for at least 20 min and survival-to-hospital-discharge ^b^Patients analyzed for time-to-first-epinephrine were limited to those with non-shockable initial rhythm ^c^Patients analyzed for time-to-defibrillation were limited to those with shockable initial rhythm ^d^The post-audit population was only included in the pre-post analysis The number of post-audit patients analyzed were lower than those eligible because the analyses were based on the cluster level. Therefore, only the patients who had cardiac arrest in the units with both pre- and post-audit arrests were analyzed Abbreviations: IHCA; in-hospital cardiac arrest

In measuring the association between actual IHCA outcomes and audit scores, a total of 483 patients were included in the analyses involving the pre-audit period of the audit cycle 2015, while 324 post-audit patients were eligible for the pre-post analyses. Their characteristics were mostly comparable to the whole cohort (Table 2). For the primary hypothesis, the whole pre-audit population was analyzed for sustained ROSC and survival-to-hospital-discharge, while the number of patients analyzed for time-to-first-epinephrine and time-to-defibrillation were 144 and 55, respectively (Fig. 2). The number of clusters and observations for each analysis is reported in the footnotes of the result tables.

### Association between audit results and actual outcomes

For sustained ROSC and survival-to-hospital-discharge, there were ≥ 50 clusters and an average of 7.2 cardiac arrests per cluster. For sustained ROSC, there was a positive association between higher audit scores and sustained ROSC with a 31–34% increase in the odds of the outcome per 1-point increase in audit score (Table 3). For survival-to-hospital-discharge, all the point estimates showed a positive association between higher audit scores and survival-to-hospital-discharge although they failed to meet statistical significance (Table 3).

**Table 3:** Association between audit scores and outcomes/arrest performance indicators in the pre-inspection period

Model	Outcome
	Return of spontaneous circulation for at least 20 min
**Null**	ICC 0.002
**1**	**cOR 1.31 (1.06, 1.62); p = 0.01**
**2**	**aOR 1.32 (1.04, 1.67); p = 0.01**
	**Survival-to-hospital-discharge**
**Null**	ICC 0.20
**1**	cOR 1.37 (0.84, 2.22); p = 0.21
**2**	N/A
	**Time-to-first-epinephrine** **for non-shockable initial rhythm**
**Null**	ICC 0.27
**1**	**Difference − 0.30 (− 0.56, − 0.05); p = 0.02** *Expected 26.2% decrease for 1 unit increase in audit score*
**2**	Difference − 0.19 (− 0.47, 0.08); p = 0.17
	**Time-to-defibrillation**
**Null**	ICC 0.17
**1**	**Difference − 0.53 (− 0.97, − 0.08); p = 0.02** *Expected 41.0% decrease for 1 unit increase in audit score*
**2**	**Difference − 0.42 (− 0.73, − 0.11); p = 0.009** *Expected 34.2% decrease for 1 unit increase in audit score*

Notes:- Data are presented as odds ratio (95%CI). Model descriptions: Null model = only a random intercept for arrest unit; Model 1 independent variables = subsequent audit score as a continuous variable with a random intercept for the arrest unit; Model 2 independent variables for return of spontaneous circulation for at least 20 min and survival to hospital discharge = subsequent audit score, the arrest ward type (emergency department, intensive care unit, ward or other), and patient characteristics including age, gender, initial shockable rhythm, end-stage renal disease, chronic kidney disease, hematologic malignancy, solid neoplasia, heart disease, and liver disease; Model 2 independent variables for time-to-first-epinephrine = subsequent audit score, the arrest ward type, and intravenous access prior to arrest, and Model 2 independent variables for time-to-defibrillation = subsequent audit score, the arrest ward type, and electrocardiogram monitoring pre-arrest. Time-to-first-epinephrine and time-to-defibrillation were log transformed for multilevel model analyses. To interpret their results on a multiplicative scale, we obtained the percentage change in the outcome by a one-unit change in the independent variable by anti-logging the beta-coefficient minus 1, followed by multiplying the product by 100

Number of clusters (hospital units) and observations for return of spontaneous circulation = 61 and 483, for survival-to-hospital-discharge = 61 and 483, for time-to-first-epinephrine = 36 and 144, for time-to-defibrillation = 25 and 55

Abbreviations: N/A, not enough clusters or observations for multilevel regression model; ICC, intraclass correlation coefficient; cOR, crude odds ratio; aOR, adjusted odds ratio

For time-to-first-epinephrine and time-to-defibrillation, there were ≤ 50 clusters and an average of 3.4 and 2.0 cardiac arrests per cluster, respectively. For time-to-first-epinephrine, all point estimates showed higher audit scores were associated with reductions in time-to-first-epinephrine, but only model 1 was significant. For time-to-defibrillation, higher audit scores were significantly associated with reductions in time-to-defibrillation (Table 3).

Sensitivity analyses showed comparable results to primary ones (Additional file 5). Also, subgroup analyses demonstrated better outcomes and performance indicators with higher audit scores were primarily achieved in the ICU (Additional file 6).

### Pre-post comparison of hospital units passing or failing an audit inspection

Hospital units failing an audit did not show improvement in both patient outcomes and arrest performance indicators. The point estimates showed lower sustained ROSC rates and increased time-to-first-epinephrine after failing although no models met statistical significance. Survival-to-hospital-discharge and time-to-defibrillation would not converge to a solution due to sparse data. For passing units, there was a significant decrease in time-to-first-epinephrine after passing while other models for other outcomes were not statistically significant (Table 4).

**Table 4:** Pre-post analyses of hospital units that failed or passed an audit

	Hospital units that *FAILED* an audit	Hospital units that *PASSED* an audit
Model	Return of spontaneous circulation for at least 20 min	
**Null**	ICC 0.05	ICC < 0.000001
**1**	cOR 0.31 (0.03, 2.99); p = 0.31	cOR 0.83 (0.61, 1.12); p = 0.22
**2**	aOR 0.61 (0.04, 9.55); p = 0.72	aOR 0.86 (0.62, 1.20); p = 0.38
	**Survival-to-hospital-discharge**	
**Null**	N/A	ICC 0.13
**1**	N/A	cOR 1.08 (0.67, 1.75); p = 0.75
**2**	N/A	N/A
	**Time-to-first-epinephrine for non-shockable initial rhythm**	
**Null**	ICC 6.13e-24	ICC 0.29
**1**	Difference 0.20 (− 1.69, 2.09); p = 0.82	**Difference − 0.19 (− 0.38, − 0.02); p = 0.03** *Expected 18.0% decrease with passing*
**2**	Difference 0.30 (− 1.79, 2.40); p = 0.74	**Difference − 0.19 (− 0.37, − 0.01); p = 0.04** *Expected 17.3% decrease with passing*
	**Time-to-defibrillation**	
**Null**	N/A	ICC 0.28
**1**	N/A	Difference 0.09 (− 0.56, 0.75); p = 0.78
**2**	N/A	Difference 0.07 (− 0.59, 0.74); p = 0.83

Notes:- Data are presented as odds ratio (95%CI). Model description: Null model = only a random intercept for the arrest unit; Model 1 independent variables = indicator variable for pre- and post-audit periods with a random intercept for the arrest unit; Model 2 independent variables for return of spontaneous circulation for at least 20 min/survival to hospital discharge = indicator variable for pre- and post-audit periods, arrest ward type (emergency department, intensive care unit, ward or other), and patient characteristics including age, gender, initial shockable rhythm, end-stage renal disease, chronic kidney disease, hematologic malignancy, solid neoplasia, heart disease, and liver disease; Model 2 independent variables for time-to-first-epinephrine = indicator variable for pre- and post-audit periods, arrest ward type, and intravenous access prior to arrest; and Model 2 independent variables for time-to-defibrillation = indicator variable for pre- and post-audit periods, arrest ward type, and electrocardiogram monitoring pre-arrest. Because time-to-first- epinephrine and time-to-defibrillation were log transformed for multilevel analyses, they are anti-logged minus 1, followed by multiplied by 100 to obtain the percentage change of the outcome per one unit change in independent variable for interpretation of a multiplicative scale

For passed units: number of clusters (hospital units) and observations for return of spontaneous circulation = 32 and 606, for survival-to-hospital-discharge = 34 and 759, for time-to-first-epinephrine = 34 and 759, for time-to-first-epinephrine = 25 and 559, for time-to-defibrillation = 10 and 49. For failed units: number of clusters and observations for return of spontaneous circulation = 5 and 16, for time-to-first-epinephrine = 4 and 12

Abbreviations: N/A, not enough clusters or observations for multilevel regression model; ICC, intraclass correlation coefficient; cOR, crude odds ratio; aOR, adjusted odds ratio

Sensitivity analyses yielded comparable results to primary ones (Additional file 7). Subgroup analyses showed a decrease in time-to-first-epinephrine in the passing units mostly occurred in wards rather than ICU (Additional file 8).

## Discussion

In this retrospective study, we found higher CPR in-situ simulation scores were associated with better patient outcomes and arrest performance indicators, namely sustained ROSC, time-to-defibrillation, and possibly time-to-first-epinephrine. However, failing hospital units did not show any improvement in any of the study outcomes with most improved outcomes achieved by passing hospital units.

Simulation has been implemented as a part of resuscitation education and training for decades. [23, 24] Because cardiac arrest resuscitation requires both individual skills and knowledge as well as optimized teamwork and effective communication, simulation-based training thus provides the optimal practicing environment. [23, 25] In-situ simulation has recently been introduced in the area of resuscitation. In the actual medical environment, learners tend to gain a more immersive learning experience. It also allows for system evaluation and better insights into team dynamics. [26] Nevertheless, there is limited available evidence regarding hospital-wide in-situ simulation for cardiac arrest resuscitation. Bently et al. reported that hospital-wide in-situ simulation of IHCA could effectively help to identify and mitigate latent safety threats. [27] Although the community of simulation-based research in the resuscitation field is growing, studies associating simulation outcomes, especially those of hospital-wide in-situ simulations, with those of real situations are lacking. [28, 29] To the best of our knowledge, the present study is the first to report the impact of hospital-wide in-situ simulation employed as a method of CPR performance evaluation on actual clinical outcomes and important arrest performance indicators.

Our results emphasize the value of this in-situ simulation as a potential tool to evaluate CPR performance in hospital units as its results were associated with actual patient outcomes. However, the audit results showed most hospital unit categories performed worse in later audit cycles except for ED. This trend could have occurred partly because there were many new OPDs and wards introduced in later years, as depicted in Table 1. These new units might have had less experience in CPR, leading to lower scores. Nevertheless, the unit categories with higher scores (ED and ICU) were those encountering more actual arrest cases. These high-risk units could also deliver better clinical outcomes in survival-to-hospital-discharge, similar to other studies of Western population. [15, 18] This result thus confirms the CPR training provided for these units was appropriate and emphasizes the importance of providing more focused training to healthcare providers from the units with higher risk of cardiac arrest events. [30] It may also indicate the audit results could have been concordant with important clinical outcomes, such as survival-to-hospital-discharge, although we could not demonstrate a significant association due to the rarity of positive outcomes. Also for this reason, we did not analyze hospital discharge with favorable neurological outcomes. The proportion of both clinical outcomes in the present study were very low compared to the reports of other registries from Western countries [31–33] but did not differ much from those of other Asian countries. [34, 35] Regardless, we believe that our worse clinical outcomes could have been because of the limited resource in providing optimal post-cardiac arrest care and neurological protective strategies in our hospital setting during the study period. Nonetheless, we chose sustained ROSC as the primary clinical outcome because it is more relevant to the CPR training and audit, which do not involve much of the post-cardiac arrest care process.

Furthermore, we found failing units could not improve as would have been anticipated after failing while passing units could improve some study outcomes, concordant with our primary hypothesis results demonstrating better performance in units with higher scores. This result highlights the need to enhance CPR training for failing units, especially those in high-risk unit-categories.

Nevertheless, it is important to note that the in-situ simulations were conducted only during office hours on weekdays, so the audit scores may not reflect night-shift or weekend CPR performance. More importantly, they were performed once in several years apart with one scenario per hospital unit per audit cycle, which may limit the implication of the audit scores derived from a group of providers to reflect the performance of the whole unit. In fact, the participants in the audit might not have been those resuscitating real patients that were analyzed. Therefore, these simulations should be conducted more often to better reflect the true performance of hospital units and thus increase the credibility of the associations with actual patient outcomes. Also, with wider coverage and higher frequency, this in-situ simulation initially aimed for evaluation purposes could also serve as a training intervention to improve CPR performance. Another issue with the in-situ simulation procedure was regarding how the results were evaluated. Although the checklist for evaluation was created based on the standard guidelines, it still relies largely on subjective decisions and needs to be further validated. Also, despite the auditors being trained and validated, it is imperative to consistently assess their inter-rater reliability. In fact, a decreasing trend of audit scores over the years was discordant with the observed annual trend of no change in ROSC or improvement in survival-to-hospital-discharge (data not shown), which may reflect an unstandardized evaluation tool and process as the discordance is contrary to expectation.

Considering all the findings together and despite its current weaknesses, we suggest an audit with in-situ simulation may be an effective method for performance evaluation of a complex procedure such as CPR. Furthermore, our analyses suggest specific areas to focus on to improve CPR training, hospital resuscitative services, and patient outcomes.

### Limitations

The present study has limitations. Firstly, this was a single-center retrospective study, which limits the generalizability of our results. Secondly, even though the evaluation tool contains items from the standard practice guidelines and its inter-rater reliability was already assessed, such analysis was done only once while no other psychometric properties have been analyzed. Inter-rater reliability should be re-examined, although the final score for each item was based on the consensus of a group of auditors and not directly based on their raw ratings. Thirdly, we did not collect the audited team characteristics because we considered CPR performance as an overall team performance and because we expect healthcare personnel working in the same unit to have undergone similar training and had comparable experiences in resuscitation. Nevertheless, our assumption might not be accurate. If some team characteristics affected both the audit score and actual patient outcomes, they should have been collected and adjusted for in the analyses. Fourthly, there were indeterminant proportions of missing data on time-to-first-epinephrine and time-to-defibrillation because of missing data on initial rhythm (7.5% missing data). Thus, the direction and magnitude of bias cannot be determined, and no missing data handling is possible. Additionally, there could have been selection bias in the pre-post analyses because only the hospital units with observations in both periods could be analyzed, which may have selected hospital units that more frequently have IHCA. Another limitation is CPR records were written as a form by healthcare providers, possibly resulting in missing or inaccurate data, especially the intervention time records. Moreover, the return rate of the audited form may not have been 100% and could limit the interpretation of the true quality of resuscitation and the effect of in-situ simulation.

## Conclusion

Results of CPR in-situ simulations appeared to reflect clinical outcomes and key arrest performance indicators, evidencing that audit inspection may have value in reflecting real-world performance. Therefore, it may be an effective method of CPR performance evaluation that can direct hospital administrators towards appropriate areas of improvement.

## Electronic supplementary material

Below is the link to the electronic supplementary material.

Additional file 1: Definitions and exclusions of arrest performance indicators.

Additional file 2: Details of the five unit-categories in the simulation-in-situ audit.

Additional file 3: The CPR audit evaluation form.

Additional file 4: Study outcomes by the arrest unit type.

Additional file 5: Association between subsequent audit score and outcomes/arrest performance indicators in the pre-audit inspection period – sensitivity analysis.

Additional file 6: Association between subsequent audit score and outcomes/arrest performance indicators in the pre-inspection period – subgroup analysis.

Additional file 7: Pre-post analyses of hospital units that failed and passed an audit – sensitivity analysis.

Additional file 8: Pre-post analyses of units that failed and passed an audit – subgroup analysis.

## Data Availability

The dataset is not available but can be requested from the corresponding author. ***Competing interests***. None.

## List of Abbreviations

IHCA: In-hospital cardiac arrest
CPR: Cardiopulmonary resuscitation
AHA: American Heart Association
ERC: European Resuscitation Council
ACLS: Advanced cardiovascular life support
BLS: Basic life support
ICU: Intensive care units
ED: Emergency departments
OPD: Outpatient units
ROSC: Return of spontaneous circulation
