# Supplementary material for: Association between cardiopulmonary resuscitation audit results with in-situ simulation and in-hospital cardiac arrest outcomes and key performance indicators
Source: BMC Cardiovasc Disord. 2023 Jun 13;23:299. doi: 10.1186/s12872-023-03320-w (PMC10265752; doi:10.1186/s12872-023-03320-w)
Supplement: Supplementary file 1 — Additional file 1: Definitions and exclusions of arrest performance indicators. [file 12872_2023_3320_MOESM1_ESM.docx]

**Definitions and exclusions of arrest performance indicators**

Time-to-first-epinephrine for non-shockable initial rhythms

Definition = duration between no pulse and first epinephrine

Exclusions = first epinephrine delivered before no pulse, hemodynamic drug support prior to arrest, and missing data or unknown initial rhythm.

**Reference**

Donnino MW, Salciccioli JD, Howell MD, Cocchi MN, Giberson B, Berg K, Gautam S, Callaway C; American Heart Association’s Get With The Guidelines-Resuscitation Investigators. Time to administration of epinephrine and outcome after in-hospital cardiac arrest with non-shockable rhythms: retrospective analysis of large in-hospital data registry. BMJ. 2014 May 20;348**:**g3028.

Time-to-defibrillation for shockable initial rhythm

Definition = duration between no pulse and first defibrillation

Exclusions = first defibrillation before no pulse, hemodynamic drug support prior to arrest, implanted defibrillator, and missing data or unknown initial rhythm.

**Reference**

Chan PS, Krumholz HM, Nichol G, Nallamothu BK; American Heart Association National Registry of Cardiopulmonary Resuscitation Investigators. Delayed time-to-defibrillation after in-hospital cardiac arrest. N Engl J Med. 2008 Jan 3;358(1):9-17.

**N.B.** We did not analyze time-to-epinephrine for shockable rhythm because of the questionable clinical importance of epinephrine for such rhythms and the time from which the duration should be defined.
