# Supplementary material for: Association between cardiopulmonary resuscitation audit results with in-situ simulation and in-hospital cardiac arrest outcomes and key performance indicators
Source: BMC Cardiovasc Disord. 2023 Jun 13;23:299. doi: 10.1186/s12872-023-03320-w (PMC10265752; doi:10.1186/s12872-023-03320-w)
Supplement: Supplementary file 2 — Additional file 2: Details of the five unit-categories in the simulation-in-situ audit. [file 12872_2023_3320_MOESM2_ESM.docx]

**Details of the five unit-categories in the simulation-in-situ audit**

For the CPR audit, the Sirriaj CPR center categorized the hospital units into five categories according to their risk of having an occurrence of cardiac arrest. These categories are as follows:

1) Emergency department (ED) and intensive care unit (ICU)

2) Critical wards; where about a third of patients are critically-ill patients to whom ICU could not be provided

3) Procedural units

4) General wards

5) Outpatient units (OPD)

In category 1, physicians and nurses are available on the floor 24 hours, and there were enough monitors, defibrillators and other resources for resuscitation for each patient. In category 2, the ratio of physicians and nurses per patient was lower than in the first. There was at least one defibrillator, emergency cart and monitoring equipment per one ward. Categories 3, 4 and 5 were covered by an on-call ACLS team who would respond to the emergency activation system of the hospital. One defibrillator-monitor is shared by two or more general wards or units. All residents, staffs and nurses working in unit category 1 through 3 were obligated to attend an ACLS course organized by the Siriraj CPR center. They also had to renew the course every three years. While the nurses working in unit category 4 and 5 had to at least pass a BLS course.
