# Supplementary material for: Association between cardiopulmonary resuscitation audit results with in-situ simulation and in-hospital cardiac arrest outcomes and key performance indicators
Source: BMC Cardiovasc Disord. 2023 Jun 13;23:299. doi: 10.1186/s12872-023-03320-w (PMC10265752; doi:10.1186/s12872-023-03320-w)
Supplement: Supplementary file 3 — Additional file 3: The CPR audit evaluation form. [file 12872_2023_3320_MOESM3_ESM.docx]

**The CPR audit evaluation form**

| **Domains and items** | **Rating options/scale range** |
| --- | --- |
| **First response reaction** | **0-10 (judged by criteria and overall performance in the domain)** |
| 1. Initial patient assessment |  |
| - Time to assessment | seconds |
| - Time to first compression | minutes |
| - Enthusiasm | Very good/good/not good |
| - Skills | Adequate/inadequate |
| 2. Basic “Circulation” skills |  |
| - Pulse assessment | Adequate/inadequate |
| - Chest compression; depth* | Always adequate/ >80% adequate/ <80% adequate |
| - Chest compression; frequency* | Average rate >120/min/rate 100-120/min/ rate <100/min |
| - Chest compression position* | Appropriate/ inappropriate |
| - Cardiac board placed | Yes/No |
| - Automated defibrillator skills | Adequate/inadequate |
| 3. Basic “Airway” & ”Breathing” skills |  |
| - Bag-mask ventilation* | Adequate/inadequate |
| - Airway opening* | Adequate/inadequate |
| - Monitoring chest movement | Adequate/inadequate |
| - High-flow oxygen attached | Yes/No |
| 4. Resuscitation equipment preparation |  |
| - Completeness | Yes/No |
| - Ready to use | Yes/No |
| - Priority of preparation | Appropriate/ inappropriate |
| **Overall ACLS skills** | **0-10 (judged by criteria and overall performance in the domain)** |
| 1. Defibrillation |  |
| - Skills | Adequate/inadequate |
| - Timing (time to defibrillation)* | Adequate/inadequate |
| - Support from the team | Adequate/inadequate |
| 2. High-quality CPR |  |
| - Endorsement/monitoring | Adequate/inadequate |
| 3. Reversible causes |  |
| - Find the causes | Adequate/inadequate |
| - Correct the causes | Adequate/inadequate  Appropriate/ inappropriate |
| 4. Algorithm diagnosis | All correct/>50% correct/<50% correct |
| 5. Medications in the algorithm |  |
| - Correctness | Appropriate/ inappropriate |
| - Timing | Appropriate/ inappropriate |
| 6. Cycle monitoring |  |
| - Resuscitation timed and called | Adequate/inadequate |
| - Patients assessed every cycle | Appropriate/ inappropriate |
| **Team dynamics** | **0-10 (judged by criteria and overall performance in the domain)** |
| 1. Clear messages | Adequate/inadequate |
| 2. Clear roles and responsibilities | Adequate/inadequate |
| 3. Constructive interventions | Adequate/inadequate |
| 4. Closed-loop communication | Adequate/inadequate |
| 5. Summarizing and re-evaluation | Adequate/inadequate |
| 6. Knowledge sharing | Adequate/inadequate |
| **Emergency activation system** | **0-10 (judged by criteria and overall performance in the domain)** |
| 1. Knowledge of the process | Adequate/inadequate |
| 2. Timing of the process | Adequate/inadequate |
| 3. Facilitate advanced team arrival | Adequate/inadequate |

* Evaluated by the monitoring program embedded in the mannequin
