# Supplementary material for: Association between cardiopulmonary resuscitation audit results with in-situ simulation and in-hospital cardiac arrest outcomes and key performance indicators
Source: BMC Cardiovasc Disord. 2023 Jun 13;23:299. doi: 10.1186/s12872-023-03320-w (PMC10265752; doi:10.1186/s12872-023-03320-w)
Supplement: Supplementary file 4 — Additional file 4: Study outcomes by the arrest unit type. [file 12872_2023_3320_MOESM4_ESM.docx]

**Supplementary Table. Study outcomes by the arrest unit type**

| **Hospital unit type** | **Return of spontaneous circulation ≥ 20 min, n (%)** | **Survival to hospital discharge, n (%)** | **Time-to-first-epinephrine for initial non-shockable rhythm, median (IQR), min** | **Time-to-defibrillation for initial shockable rhythm, median (IQR), min** |
| --- | --- | --- | --- | --- |
| **All** | **n=2146** | **n=2146** | **n=1571** | **n=188** |
|  | 1402 (65.3) | 276 (12.9) | 1 (0, 3) | 3 (1, 5) |
| **ED** | **n=383** | **n=384** | **n=305** | **n=26** |
|  | 243 (63.3) | 75 (19.5) | 1 (1, 2) | 2 (0, 3) |
| **ICU** | **n=681** | **n=681** | **n=425** | **n=84** |
|  | 468 (68.7) | 96 (14.1) | 1 (0, 3) | 2 (0.5, 5) |
| **Ward** | **n=1052** | **n=1052** | **n=820** | **n=76** |
|  | 670 (63.7) | 96 (9.1) | 2 (0, 5) | 5 (2.5, 10) |
| **Other** | **n=29** | **n=29** | **n=21** | **n=2** |
|  | 21 (72.4) | 9 (31.0) | 5 (2, 7) | 4 (3, 5) |

Abbreviations: ED, emergency department; ICU, intensive care unit; IQR, interquartile range
