# Supplementary material for: Association between cardiopulmonary resuscitation audit results with in-situ simulation and in-hospital cardiac arrest outcomes and key performance indicators
Source: BMC Cardiovasc Disord. 2023 Jun 13;23:299. doi: 10.1186/s12872-023-03320-w (PMC10265752; doi:10.1186/s12872-023-03320-w)
Supplement: Supplementary file 5 — Additional file 5: Association between subsequent audit score and outcomes/arrest performance indicators in the pre-audit inspection period – sensitivity analysis. [file 12872_2023_3320_MOESM5_ESM.docx]

**Supplementary Table. Association between subsequent audit score and outcomes/arrest performance indicators in the pre-audit inspection period – sensitivity analysis**

| **Model** | **All units excluding 1 month before audit^a^** | **Wards and intensive care units only^b^** |
| --- | --- | --- |
|  | **Return of spontaneous circulation for at least 20 minutes** | |
| **Null** | ICC < 0.000001 | ICC 0.004 |
| **1** | **cOR 1.42 (1.13, 1.80); p=0.003** | **cOR 1.34 (1.07, 1.67); p=0.01** |
| **2** | **aOR 1.44 (1.12, 1.84); p=0.004** | **aOR 1.31 (1.04, 1.66); p=0.02** |
|  | **Survival to hospital discharge** | |
| **Null** | ICC 0.18 | ICC 0.17 |
| **1** | cOR 1.29 (0.78, 2.16); p=0.32 | cOR 1.39 (0.84, 2.30); p=0.20 |
| **2** | N/A | N/A |
|  | **Time-to-first-epinephrine** | |
| **Null** | ICC 0.12 | ICC 0.27 |
| **1** | **Difference - 0.30 (- 0.55, - 0.06); p=0.01**  *Expected 26.5% decrease for 1 unit increase in audit score* | **Difference - 0.30 (- 0.58, - 0.03); p=0.03**  *Expected 26.2% decrease for 1 unit increase in audit score* |
| **2** | Difference - 0.21 (- 0.47, 0.05); p=0.11 | Difference - 0.20 (- 0.47, 0.07); p=0.15 |
|  | **Time-to-defibrillation** | |
| **Null** | ICC 0.16 | ICC 0.17 |
| **1** | **Difference - 0.62 (- 1.07, - 0.18); p=0.006**  *Expected 46.4% decrease for 1 unit increase in audit score* | Difference - 0.53 (- 1.07, 0.03); p=0.05 |
| **2** | **Difference - 0.41 (- 0.73, - 0.10); p=0.009**  *Expected 33.9% decrease for 1 unit increase in audit score* | **Difference - 0.42 (- 0.73, - 0.11); p=0.009**  *Expected 34.2% decrease for 1 unit increase in audit score* |

Notes:- Data are presented as odds ratio (95%CI). Model descriptions: Null model = only a random intercept for arrest unit; Model 1 independent variables = subsequent audit score as a continuous variable with a random intercept for the arrest unit; Model 2 independent variables for return of spontaneous circulation for at least 20 minutes and survival to hospital discharge = subsequent audit score, the arrest ward type (emergency department, intensive care unit, ward or other), and patient characteristics including age, gender, initial shockable rhythm, end-stage renal disease, chronic kidney disease, hematologic malignancy, solid neoplasia, heart disease, and liver disease; Model 2 independent variables for time-to-first-epinephrine = subsequent audit score, the arrest ward type, and intravenous access prior to arrest, and Model 2 independent variables for time-to-defibrillation = subsequent audit score, the arrest ward type, and electrocardiogram monitoring pre-arrest. Time-to-first-epinephrine and time-to-defibrillation were log transformed for multilevel model analyses. To interpret their results on a multiplicative scale, we obtained the percentage change in the outcome by a one-unit change in the independent variable by anti-logging the beta-coefficient minus 1, followed by multiplying the product by 100.

^a^Number of clusters and observations for return of spontaneous circulation = 57 and 442, for survival to hospital discharge = 57 and 442, for time-to-first-epinephrine = 35 and 126, for time-to-defibrillation = 22 and 51.

^b^Number of clusters and observations for return of spontaneous circulation = 54 and 445, for survival to hospital discharge = 54 and 445, for time-to-first-epinephrine = 33 and 132, for time-to-defibrillation = 23 and 52.

Abbreviations: N/A, not enough clusters or observations for multilevel regression model; ICC, intraclass correlation coefficient; cOR, crude odds ratio; aOR, adjusted odds ratio
