# Supplementary material for: Association between cardiopulmonary resuscitation audit results with in-situ simulation and in-hospital cardiac arrest outcomes and key performance indicators
Source: BMC Cardiovasc Disord. 2023 Jun 13;23:299. doi: 10.1186/s12872-023-03320-w (PMC10265752; doi:10.1186/s12872-023-03320-w)
Supplement: Supplementary file 6 — Additional file 6: Association between subsequent audit score and outcomes/arrest performance indicators in the pre-inspection period – subgroup analysis. [file 12872_2023_3320_MOESM6_ESM.docx]

**Supplementary Table. Association between subsequent audit score and outcomes/arrest performance indicators in the pre-inspection period – subgroup analysis**

| **Model** | **Emergency departments^a^** | **Intensive care units^b^** | **Wards^c^** |
| --- | --- | --- | --- |
|  | **Return of spontaneous circulation for at least 20 minutes** | | |
| **Null** | N/A | ICC 0.03 | ICC < 0.000001 |
| **1** | N/A | **cOR 1.43 (1.02, 2.02); p=0.04** | cOR 1.24 (0.91, 1.70); p=0.18 |
| **2** | N/A | aOR 1.38 (0.97, 1.96); p=0.07 | aOR 1.21 (0.88, 1.67); p=0.24 |
|  | **Survival to hospital discharge** | | |
| **Null** | N/A | ICC 0.29 | ICC 0.10 |
| **1** | N/A | cOR 1.24 (0.51, 3.01); p=0.64 | cOR 1.20 (0.59, 2.42); p=0.61 |
| **2** | N/A | N/A | N/A |
|  | **Time-to-first-epinephrine** | | |
| **Null** | ICC 6.18e-25 | ICC 3.31e-27 | ICC 0.40 |
| **1** | N/A | Difference 0.03 (- 0.18, 0.24); p=0.79 | Difference - 0.29 (- 0.62, 0.04); p=0.08 |
| **2** | N/A | Difference - 0.05 (- 0.25, 0.15); p=0.65 | Difference - 0.29 (- 0.61, 0.02); p=0.07 |
|  | **Time-to-defibrillation** | | |
| **Null** | N/A | ICC 0.29 | ICC 2.31e-25 |
| **1** | N/A | **Difference - 0.63 (- 0.86, - 0.39); p<0.0001**  *Expected 46.6% decrease for 1 unit increase in audit score* | Difference 0.50 (- 0.49, 1.49); p=0.32 |
| **2** | N/A | **Difference - 0.63 (- 0.86, - 0.39); p<0.0001**  *Expected 46.6% decrease for 1 unit increase in audit score* | Difference 0.47 (- 0.44, 1.39); p=0.31 |

Notes:- Data are presented as odds ratio (95%CI). Model descriptions: Null model = only a random intercept for arrest unit; Model 1 independent variables = subsequent audit score as a continuous variable with a random intercept for the arrest unit; Model 2 independent variables for return of spontaneous circulation for at least 20 minutes and survival to hospital discharge = subsequent audit score, the arrest ward type (emergency department, intensive care unit, ward or other), and patient characteristics including age, gender, initial shockable rhythm, end-stage renal disease, chronic kidney disease, hematologic malignancy, solid neoplasia, heart disease, and liver disease; Model 2 independent variables for time-to-first-epinephrine = subsequent audit score, the arrest ward type, and intravenous access prior to arrest, and Model 2 independent variables for time-to-defibrillation = subsequent audit score, the arrest ward type, and electrocardiogram monitoring pre-arrest. Time-to-first-epinephrine and time-to-defibrillation were log transformed for multilevel model analyses. To interpret their results on a multiplicative scale, we obtained the percentage change in the outcome by a one-unit change in the independent variable by anti-logging the beta-coefficient minus 1, followed by multiplying the product by 100.

^a^Number of clusters and observations for return of spontaneous circulation = 2 and 31, for survival to hospital discharge = 2 and 31.

^b^Number of clusters and observations for return of spontaneous circulation = 14 and 169, for survival to hospital discharge = 14 and 169, for time-to-first-epinephrine = 9 and 42, for time-to-defibrillation = 8 and 26.

^c^Number of clusters and observations for return of spontaneous circulation = 40 and 276, for survival to hospital discharge = 40 and 276, for time-to-first-epinephrine = 24 and 90, for time-to-defibrillation = 15 and 26.

Abbreviations: N/A, not enough clusters or observations for multilevel regression model; ICC, intraclass correlation coefficient; cOR, crude odds ratio; aOR, adjusted odds ratio
