# Supplementary material for: Association between cardiopulmonary resuscitation audit results with in-situ simulation and in-hospital cardiac arrest outcomes and key performance indicators
Source: BMC Cardiovasc Disord. 2023 Jun 13;23:299. doi: 10.1186/s12872-023-03320-w (PMC10265752; doi:10.1186/s12872-023-03320-w)
Supplement: Supplementary file 7 — Additional file 7: Pre-post analyses of hospital units that failed and passed an audit – sensitivity analysis. [file 12872_2023_3320_MOESM7_ESM.docx]

**Supplementary Table. Pre-post analyses of hospital units that failed and passed an audit – sensitivity analysis**

|  | **Excluding 6 months after the audit date^a^** | | **wards and intensive care units only^b^** | |
| --- | --- | --- | --- | --- |
|  | **Units that *FAILED* an audit** | **Units that *PASSED* an audit** | **Units that *FAILED* an audit** | **Units that *PASSED* an audit** |
|  | **Return of spontaneous circulation for at least 20 minutes** | | | |
| **Null** | N/A | ICC < 0.000001 | ICC < 0.000001 | ICC < 0.000001 |
| **1** | N/A | cOR 0.84 (0.57, 1.24); p=0.38 | cOR 0.30 (0.03, 2.76); p=0.29 | cOR 0.81 (0.57, 1.14); p=0.23 |
| **2** | N/A | aOR 0.83 (0.54, 1.29); p=0.41 | aOR 0.61 (0.04, 9.55); p=0.72 | aOR 0.83 (0.58, 1.20); p=0.32 |
|  | **Survival to hospital discharge** | | | |
| **Null** | N/A | ICC 0.12 | N/A | ICC 0.11 |
| **1** | N/A | cOR 0.97 (0.52, 1.83); p=0.93 | N/A | cOR 1.18 (0.67, 2.05); p=0.57 |
| **2** | N/A | N/A | N/A | N/A |
|  | **Time-to-first-epinephrine** | | | |
| **Null** | N/A | ICC 0.15 | ICC 1.62e-23 | ICC 0.26 |
| **1** | N/A | Difference - 0.19 (- 0.43, 0.05); p=0.12 | 0.27 (- 2.12, 2.66); p=0.79 | **Difference - 0.27 (- 0.47, - 0.07); p=0.009**  *Expected 23.5% decrease with passing* |
| **2** | N/A | Difference - 0.18 (- 0.41, 0.06); p=0.15 | 0.20 (-2.36, 2.76); p= 0.85 | **Difference - 0.26 (- 0.46, - 0.06); p=0.01**  *Expected 23.0% decrease with passing* |
|  | **Time-to-defibrillation** | | | |
| **Null** | N/A | ICC 2.05e-23 | N/A | ICC 0.37 |
| **1** | N/A | Difference - 0.41 (- 1.71, 0.90); p=0.50 | N/A | Difference 0.01 (- 0.67, 0.70); p=0.97 |
| **2** | N/A | Difference - 0.73 (- 1.74, 0.27); p=0.14 | N/A | Difference - 0.10 (- 0.79, 0.60); p=0.78 |

Notes:- Data are presented as odds ratio (95%CI). Model description: Null model = only a random intercept for the arrest unit; Model 1 independent variables = indicator variable for pre- and post-audit periods with a random intercept for the arrest unit; Model 2 independent variables for return of spontaneous circulation for at least 20 minutes/survival to hospital discharge = indicator variable for pre- and post-audit periods, arrest ward type (emergency department, intensive care unit, ward or other), and patient characteristics including age, gender, initial shockable rhythm, end-stage renal disease, chronic kidney disease, hematologic malignancy, solid neoplasia, heart disease, and liver disease; Model 2 independent variables for time-to-first-epinephrine = indicator variable for pre- and post-audit periods, arrest ward type, and intravenous access prior to arrest; and Model 2 independent variables for time-to-defibrillation = indicator variable for pre- and post-audit periods, arrest ward type, and electrocardiogram monitoring pre-arrest. Because time-to-first- epinephrine and time-to-defibrillation were log transformed for multilevel analyses, they are anti-logged minus 1, followed by multiplied by 100 to obtain the percentage change of the outcome per one unit change in independent variable for interpretation of a multiplicative scale.

^a^For passed units: number of clusters and observations for return of spontaneous circulation = 27 and 574, for survival to hospital discharge = 27 and 574, for time-to-first-epinephrine = 24 and 432, for time-to-defibrillation = 5 and 27.

^b^For passed units: number of clusters and observations for return of spontaneous circulation = 32 and 606, for survival to hospital discharge = 32 and 606, for time-to-first-epinephrine = 28 and 459, for time-to-defibrillation = 9 and 37. For failed units: number of clusters and observations for return of spontaneous circulation = 4 and 14, for time-to-first-epinephrine = 3 and 10.

Abbreviations: N/A, not enough clusters or observations for multilevel regression model; ICC, intraclass correlation coefficient; cOR, crude odds ratio; aOR, adjusted odds ratio
