# Supplementary material for: Association between cardiopulmonary resuscitation audit results with in-situ simulation and in-hospital cardiac arrest outcomes and key performance indicators
Source: BMC Cardiovasc Disord. 2023 Jun 13;23:299. doi: 10.1186/s12872-023-03320-w (PMC10265752; doi:10.1186/s12872-023-03320-w)
Supplement: Supplementary file 8 — Additional file 8: Pre-post analyses of units that failed and passed an audit – subgroup analysis. [file 12872_2023_3320_MOESM8_ESM.docx]

**Supplementary Table. Pre-post analyses of units that failed and passed an audit – subgroup analysis**

|  | **Intensive care units^a^** | | **Wards^b^** | |
| --- | --- | --- | --- | --- |
|  | **Units that *FAILED* an audit** | **Units that *PASSED* an audit** | **Units that *FAILED* an audit** | **Units that *PASSED* an audit** |
|  | **Return of spontaneous circulation for at least 20 minutes** | | | |
| **Null** | ICC < 0.000001 | ICC < 0.000001 | ICC < 0.000001 | ICC < 0.000001 |
| **1** | N/A | cOR 0.67 (0.39, 1.16); p=0.15 | cOR 0.30 (0.03, 2.76); p=0.29 | cOR 0.92 (0.58, 1.45); p=0.71 |
| **2** | N/A | aOR 0.74 (0.42, 1.30); p=0.29 | aOR 0.61 (0.04, 9.55); p=0.72 | N/A |
|  | **Survival to hospital discharge** | | | |
| **Null** | N/A | ICC 0.03 | N/A | ICC 0.13 |
| **1** | N/A | cOR 1.67 (0.81, 3.46); p=0.17 | N/A | cOR 0.71 (0.29, 1.73); p=0.45 |
| **2** | N/A | N/A | N/A | N/A |
|  | **Time-to-first-epinephrine** | | | |
| **Null** | N/A | ICC 2.68e-19 | ICC 1.62e-23 | ICC 0.27 |
| **1** | N/A | Difference - 0.23 (- 0.58, 0.13); p=0.21 | 0.27 (- 2.12, 2.66); p=0.79 | **Difference** **- 0.30 (- 0.54, - 0.05); p=0.02**  Expected 25.8% decrease *with passing* |
| **2** | N/A | Difference - 0.24 (- 0.60, 0.12); p=0.19 | 0.20 (- 2.36, 2.76); p=0.85 | **Difference** **- 0.30 (- 0.54, - 0.05); p=0.02**  Expected 25.8% decrease *with passing* |
|  | **Time-to-defibrillation** | | | |
| **Null** | N/A | ICC 0.31 | N/A | ICC 0.34 |
| **1** | N/A | Difference 0.57 (- 0.28, 1.42); p=0.18 | N/A | Difference - 0.48 (- 1.76, 0.81); p=0.41 |
| **2** | N/A | Difference 0.47 (- 0.43, 1.37); p=0.29 | N/A | Difference - 0.48 (- 1.76, 0.81); p=0.41 |

Notes:- Data are presented as odds ratio (95%CI). Model description: Null model = only a random intercept for the arrest unit; Model 1 independent variables = indicator variable for pre- and post-audit periods with a random intercept for the arrest unit; Model 2 independent variables for return of spontaneous circulation for at least 20 minutes/survival to hospital discharge = indicator variable for pre- and post-audit periods, arrest ward type (emergency department, intensive care unit, ward or other), and patient characteristics including age, gender, initial shockable rhythm, end-stage renal disease, chronic kidney disease, hematologic malignancy, solid neoplasia, heart disease, and liver disease; Model 2 independent variables for time-to-first-epinephrine = indicator variable for pre- and post-audit periods, arrest ward type, and intravenous access prior to arrest; and Model 2 independent variables for time-to-defibrillation = indicator variable for pre- and post-audit periods, arrest ward type, and electrocardiogram monitoring pre-arrest. Because time-to-first- epinephrine and time-to-defibrillation were log transformed for multilevel analyses, they are anti-logged minus 1, followed by multiplied by 100 to obtain the percentage change of the outcome per one unit change in independent variable for interpretation of a multiplicative scale.

^a^For passed units: number of clusters and observations for return of spontaneous circulation = 11 and 247, for survival to hospital discharge = 11 and 247, for time-to-first-epinephrine = 10 and 157, for time-to-defibrillation = 4 and 23.

^b^For passed units: number of clusters and observations for return of spontaneous circulation = 21 and 359, for survival to hospital discharge = 21 and 359, for time-to-first-epinephrine = 18 and 302, for time-to-defibrillation = 5 and 14. For failed units: number of clusters and observations for return of spontaneous circulation = 4 and 14, for time-to-first-epinephrine = 3 and 10.

Abbreviations: N/A, not enough clusters or observations for multilevel regression model; ICC, intraclass correlation coefficient; cOR, crude odds ratio; aOR, adjusted odds ratio
